# Scandium and Titanium Containing Single-Walled Carbon Nanotubes for Hydrogen Storage: a Thermodynamic and First Principle Calculation

**DOI:** 10.1038/srep27370

**Published:** 2016-06-15

**Authors:** Michael Mananghaya, Dennis Yu, Gil Nonato Santos, Emmanuel Rodulfo

**Affiliations:** 1 De La Salle University, 2401 Taft Avenue, 0922 Manila, Philippines; 2 DOST-ASTHRDP; PCIEERD, Gen. Santos Ave., Bicutan, Taguig City 1631, Philippines; 3 FEU Institute of Technology, P. Paredes Street, Sampaloc, Manila; FEU East Asia College, Nicanor Reyes Street, Sampaloc, Manila, Philippines.

## Abstract

The generalized gradient approximation (GGA) to density functional theory (DFT) calculations indicate that the highly localized states derived from the defects of nitrogen doped carbon nanotube with divacancy (4ND-CN_x_NT) contribute to strong Sc and Ti bindings, which prevent metal aggregation. Comparison of the H_2_ adsorption capability of Sc over Ti-decorated 4ND-CN_x_NT shows that Ti cannot be used for reversible H_2_ storage due to its inherent high adsorption energy. The Sc/4ND-CN_x_NT possesses favorable adsorption and consecutive adsorption energy at the local-density approximation (LDA) and GGA level. Molecular dynamics (MD) study confirmed that the interaction between molecular hydrogen and 4ND-CN_x_NT decorated with scandium is indeed favorable. Simulations indicate that the total amount of adsorption is directly related to the operating temperature and pressure. The number of absorbed hydrogen molecules almost logarithmically increases as the pressure increases at a given temperature. The total excess adsorption of hydrogen on the (Sc/4ND)_10_-CN_x_NT arrays at 300 K is within the range set by the department of energy (DOE) with a value of at least 5.85 wt%.

Current echo political issues related to fossil-fuel energy resources have prompted broad interests in clean alternative energy. Biodiesel, solar energy, and wind farms are being pursued as supplements at commercially affordable scales. For automotive uses, hydrogen energy has been considered an ideal substitute for gasoline as it is recyclable and non-polluting. High-pressurized tank, liquefied form, or solid phase materials have been suggested and tested for hydrogen storage systems. Yet, none of the candidates suffices the requirements for commercial use in vehicles. Either the storage capacity or operating conditions are far short of the standard set by the Department of Energy (DOE) of the United States. Specific targets for automotive uses are summarized as gravimetric capacity of 5.5 wt% or higher for room temperature operations. Developing appropriate storage media is of the importance for practical application of hydrogen energy. Hydrogen has long been considered as a clean, abundant and efficient energy carrier.

Carbon nanotubes (CNT) have attracted much interest due to their many exceptional properties^1^,^2^,^3^,^4^,^5^,^6^,^7^,^8^,^9^,^10^,^11^,^12^,^13^,^14^,^15^,^16^,^17^,^18^,^19^,^20^, for example an extremely large surface area. Finding efficient methods of metal dispersion will be of great practical importance for developing CNT-based hydrogen storage. Unfortunately, it was demonstrated before that the hydrogen storage capacity of porous CNT at ambient temperature is no more than 1 wt%^9^. As an earth-abundant element, nitrogen is widely applied for hydrogen storage with its chemical hydrides and nanostructure forms^9^,^10^,^11^,^12^,^13^,^14^,^15^,^16^,^17^,^18^,^19^,^20^. The formation of porphyrin-like (4ND)^9^,^10^ vacancy structures can alter the chemical and physical properties of CNT. It was suggested as an origin for the acceptor like states which are very crucial for enhancing the metal binding to the defects. The 4ND vacancy is characterized by a four-nitrogen divacancy formed by removing two C atom among hexagons and replacing the four surrounding C atoms with four N atoms. The Nitrogen atoms inherently present in 4ND doping in graphitic carbon materials such as CNT have been reported to generate acceptor like states contrary to common perceptions^10^,^11^,^12^,^13^,^14^,^15^,^16^.

The transition metals (TMs)-dispersed materials have been studied recently for large hydrogen storing capacity with respect to release temperature the TM-H_2_ binding energy and ratio look very promising. However, the issue of structural stability and poor reversibility in TM dispersion has been a major concern as TM atoms tend to easily aggregate instead of being atomistically dispersed^10^,^12^,^13^. Strong metal cohesion is believed to be responsible for the aggregation. As a way to overcome such clustering, it was suggested to increase the binding strength between metal and dispersant materials (e.g., carbon based nanostructures) by introducing structural or chemical defects^10^,^11^. For example, nitrogen doping in graphitic materials was shown to improve dramatically the metal dispersion and hydrogen adsorption^11^. The 4ND defects in N-doped nanostructures enhance the reactivity and immobilization of TM^18^,^20^. To avoid the metal atoms forming cluster on the CNT, the metal species should meet the requirement that the binding energies are higher than their corresponding crystalline cohesive energies (E_coh_)^20^. Among the 3d block TMs the Sc, Ti, V, Fe, Co and Ni show higher binding energies than their cohesive energies. However, only Scandium, Titanium and Vanadium qualify for efficient hydrogen storage at ambient conditions wherein the ideal adsorption energy should be at least 0.16 eV/H_2_ for realizable reversible adsorption and desorption^20^ with promising system-weight efficiency. The CNT with 4ND functionalized with Sc, Ti, and V are seen as an excellent hydrogen storage media with five, four, four H_2_ adsorbed, respectively per metal as predicted by the famous 18-electron rule and they are lightweight.

Previous computations on hydrogen storage^16^,^17^ identified that Sc, Ti, V are appropriate TM adsorbate for CNT surface addressing adsorption stability and increase in hydrogen storage capacity. In this paper, Nitrogen doped Carbon Nanotube with divacancy (4ND-CN_x_NT) as an effective medium for Sc and Ti atomic dispersion is elucidated in detail. Vanadium functionalized 4ND-CN_x_NT is currently being investigated and other metals aside from the 3d block TMs in which the results will be published elsewhere^20^. So which of the two TM candidates mentioned would be the best adsorbate for 4ND-CN_x_NT as hydrogen storage material with respect to stability and hydrogen storage capacity at ambient room temperature? Here we perform Density Functional Theory (DFT) calculations on the formation energy of the (4ND)_n_-CN_x_NT with n = 1 to 10 4ND deficiencies. A thorough comparison of the possibility that Sc and Ti decorated 4ND-CN_x_NT behave as an ideal H_2_-storage material was evaluated in detail. These was achieved by examining the binding energies as the number of H_2_ molecules gradually increased from 1 to 5 for Sc, 1 to 4 for Ti. A model for the dispersion of the best metal atom to the 4ND_10_-CN_x_NT complexes with respect to the largest number of H_2_ adsorption that is preferentially reversible was generated to fully simulate the adsorption scenario.

To gain additional insight into the microscopic details of the adsorption process, molecular modeling using central force fields is included in the manuscript to explain bulk properties of hydrogen storage and to provide adequate background information for theoretical and experimental practitioners. The condensed-phase optimized molecular potentials for atomistic simulation studies (COMPASS)^21^,^22^ force field was directly applied to system under study to explore the microscopic properties, dependence of molecular adsorption with respect to temperature and pressure. More specifically, the good agreement with experiment using COMPASS in simulating the adsorption of several molecules adsorbed on carbon warrants its use^22^. In the succeeding work illustrated below, the COMPASS force field determines the physisorbed state of H_2_ on 4ND_10_-CN_x_NT metal complexes with respect to the largest number of H_2_ adsorbed at temperatures starting from 77 K, the normal hydrogen adsorption process for the condition of liquid nitrogen at about 0.015 Gpa, to 400 K. The long-term objective is to aid in the development of accurate and reliable descriptions on the physical properties of molecular adsorption of H_2_ on metal/4ND_10_-CN_x_NT surfaces that are capable of reversibly storing hydrogen at high densities within DOE specifications.

## Computational

**Figure 1:**
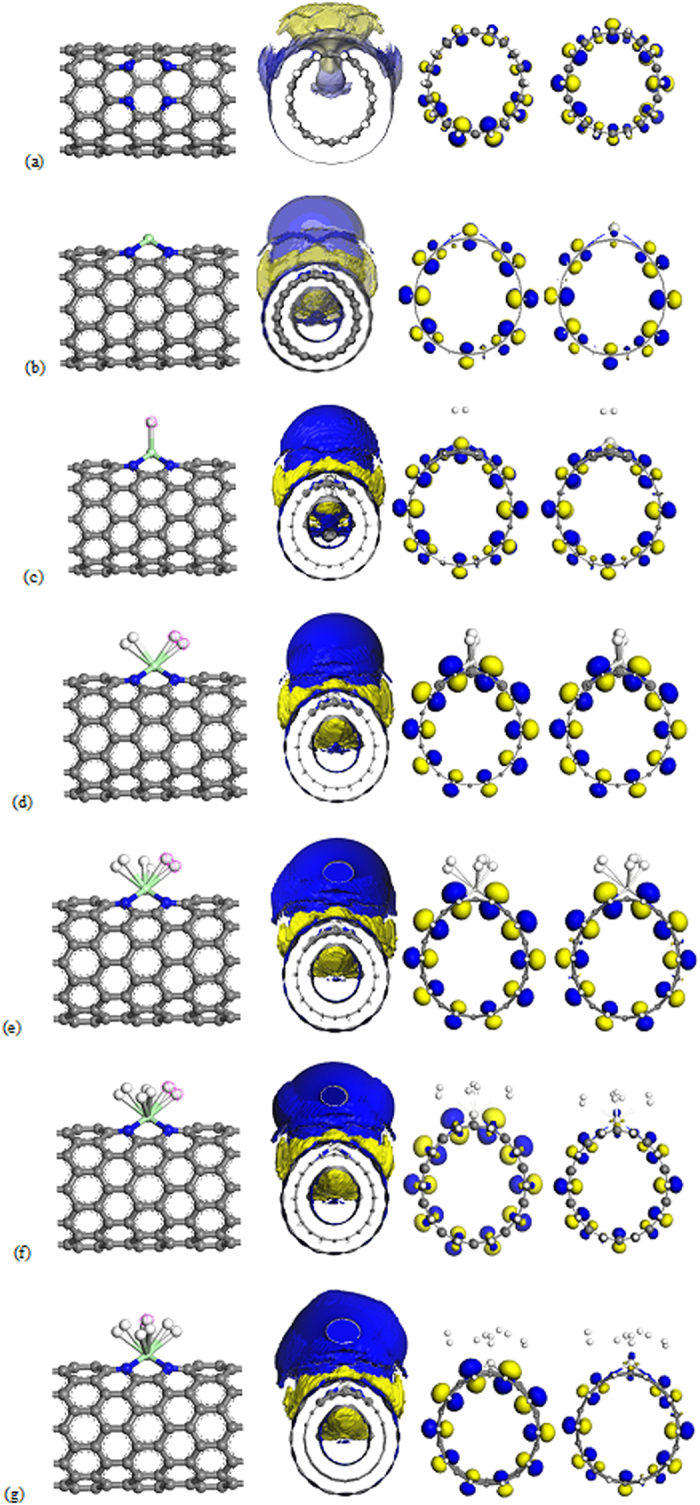
Structure, Surface Electrostatic Potential Map (SEP) and HOMO-LUMO of the optimized (**a**) 4ND-CN_x_NT, (**b**) Sc/4ND-CN_x_NT and Sc/4ND-CN_x_NT with (**c**) H_2_, (**d**) 2H_2_, (**e**) 3H_2_, (**f**) 4H_2_, (**g**) 5H_2_ systems. Gray color depicts Carbon; blue is Nitrogen; green is Scandium and white is Hydrogen.

The 4ND-CN_x_NT was constructed as shown in Fig. 1(a). The DFT calculations are carried out by the Dmol3 code from Accelrys^21^. The generalized gradient-corrected Perdew-Burke-Ernzerhof (PBE/GGA) functional^23^, along with a double numerical basis set including p-polarization function (DNP), is applied for the geometry optimization and property calculations. Dispersion-corrected DFT (DFT-D) scheme is used to describe the van der Waals (vdW) interaction. DFT semi-core pseudo-potentials (DSPPs) were employed to efficiently treat the core electron of the Sc and Ti metals. The incorporation of DFT-D scheme further improves the accuracy in evaluating weak interactions wherein the geometry optimizations are carried out using the Broyden-Fletcher-Goldfarb-Shanno (BFGS) algorithm with convergence threshold values specified as 1 × 10^−5^ Ha for energies, 2 × 10^−3^ Ha/Å for gradient and 5 × 10^−3^ Å for displacement.

The Forcite algorithm is an iterative calculation available also from Accelrys^21^ used for the molecular dynamics simulations in this paper. Due to its relatively low computational cost in comparison to DFT formalism, the COMPASS force field^22^ was selected to study larger systems. The COMPASS by definition possesses two portions: one that deals with the valence terms and the other that addresses the nonbonded interaction terms, which represent most of the energetic contributions of a physisorbed H_2_ molecule. The functional form for the nonbonded contributions includes a Lennard-Jones term and a Coulombic term which account for the vdW and the electrostatic interactions, respectively. The nonbonded energies were computed via Ewald summation wherein successful application in several investigations of carbon-based materials at the atomic scale is already established^24^. To achieve a reasonable three-dimensional model the optimized structure was redefined to create a supercell with a = b = 136.84 Å and c = 200.52 Å. During simulations, the calculate bonds tool monitors bonds, and automatically recalculates bonds if the position of the atoms changes. Typical NVT coupled to a NooséHover thermostat run time for all our simulations were 2.5 ns with time steps of 0.1 fs^25^. Different simulation temperatures from 77 up to 400 K were considered in order to determine the influence of temperature on the hydrogenation dynamics.

## Results and Discussions

### H_2_ adsorption of Sc decorated 4ND-CN_x_NT

The atomic structure of the 4ND-CN_x_NT shows that the four nitrogen atoms within a unit cell have lone pairs of electrons that form highly localized acceptor like states near the Fermi level, as shown in Fig. 1(a). This acceptor like states led to a stronger binding of Sc atoms to the absorbent than pure CNT. The calculated charge transfer using population analysis shows that the charges transferred from Sc atom to CNT in this case carries a positive charge of 0.704 e. Partially cationic character of the Sc as shown in Fig. 1(b) is due to the charge transfer from metal to the (10, 0) 4ND-CN_x_NT and thus facilitating the adsorption of foreign molecules such as hydrogen gas^20^. It has been known that the charge transfer between TM atoms and ligands profoundly affect the H_2_ binding property^11^,^26^. For example, the strong Sc binding to the 4ND-CN_x_NT leads to different characteristics of H_2_ adsorption on 4ND-CN_x_NT from those on pure CNT. To study this issue in detail, the system is modified by incrementally attaching H_2_ as one H_2_ molecule is adsorbed on the TM/4ND-CN_x_NT system the H-H bond length is elongated. Figure 1(c) shows the structural variation from spin polarized calculations as a single H_2_ molecule approaches Sc/4ND-CN_x_NT system. The energy first decreases slowly as the hydrogen gets closer to the nanotube and Sc atom. However, as the charge overlap gets large, the H_2_ molecule is attracted towards the atom with a sudden decrease in the energy. For the Sc/4ND-CN_x_NT case, the optimized Sc-H and Sc-N distances are found to be 2.324 and 2.145 Å on the average, respectively. At this point, the H_2_ molecule is still intact with a slightly increased H-H bond length from 0.752 Å of a free H_2_ to 0.766 Å due to the charge transfer from the H_2_ molecules to the Sc/4ND-CN_x_NT. The Sc/4ND-CN_x_NT has an initial adsorption of 0.279 eV/H_2_. The H_2_ adsorption energies E_ads_ in 4ND-CN_x_NT decorated by Sc was calculated by
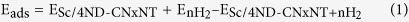


where E_Sc/4ND-CNxNT+_ _nH2_, E_Sc/4ND-CNxNT_ and E_nH2_ are the total energies of the Sc-decorated 4ND-CN_x_NT with n H_2_ molecule, Sc-decorated 4ND-CN_x_NT and n H_2_ molecule adsorbed, respectively. The calculated adsorption energies for n H_2_ is summarized in Table S1 together with the other parameters for maximal n H_2_ adsorption. The E_ads_ in the Sc-4ND-CN_x_NT complex are larger than those in Sc^+^ pure CNT complex^27^,^28^,^29^,^30^. Remarkably, it is also energetically favorable for the group to complex with additional hydrogen molecules as shown in Fig. 1(d–g) where the optimized structure, Surface Electrostatic Potential Map (SEP) and Highest Occupied Molecular Orbital-Lowest Unoccupied Molecular Orbital (HOMO-LUMO)^31^ variation is displayed as the succeeding four molecules approach the Sc atom attached to its first hydrogen, respectively. Just like the first adsorption, the succeeding four hydrogen molecules are in intact but with a rather elongated bond length of 0.758, 0.757, 0.755, 0.755 Å. For the complete structural parameters please refer to Figure S1. This 5% increase is rather a reminiscent of the elongated H-H bonds complexes first synthesized by Kubas^32^,^33^. The Orbitals showing σ-donation from H_2_ to the Sc metal and π-back-donation from the metal to the dihydrogen dominates, where Sc atoms-in-4ND-CN_x_NT analysis indicates that the electron density at the bond critical points of the bound H_2_ is similar to that of classical Kubas systems. It is found that after the adsorption for the first H_2_ the Sc still carries a positive charge of 0.567 e, indicating that more H_2_ molecules can be absorbed where the GGA predicted that for the Sc atom case it can absorb up to 5 H_2_. The analysis on the Sc-H_2_ distance reveals that the first added H_2_ is particularly affected by the 4 surrounding H_2_ molecules such that the first H_2_ molecule of 5 H_2_ molecules adsorbed is closer to the Sc atom. For a single H_2_ on Sc, the first H_2_ molecule exhibits comparable adsorption energy with respect to the following H_2_ molecules adsorbed. Addition of the second to fifth H_2_ molecule gains energies within 0.146–0.223 eV per H_2_, and they are adsorbed around the Sc as shown in Fig. 1(g) with the first H_2_ situated at the center. Here all of the five hydrogen molecules stay intact and benefit equally from bonding with the Sc atom with each molecule possessing an excess charge of about 0.520 e. The fifth H_2_ adsorption to Sc metal is 0.146 eV/H_2_, which suggests that the bonding in both Sc is an unusual combination of chemisorption and physisorption.

For efficient hydrogen storage at ambient conditions, the consecutive adsorption energy (ΔE) should be in the range of 0.16–0.42 eV/H_2_ intermediate between physiosorption and chemisorptions for realizable reversible adsorption and desorption^20^,^30^,^34^,^35^,^36^. The ΔE is calculated based on the following formula


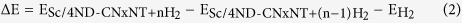


where E_Sc/4ND-CNxNT+_ _nH2_ and E_Sc/4ND-CNxNT+_ _(n−1)H2_ are the total energies of Sc-decorated 4ND-CN_x_NT with n and n − 1 H_2_ molecules, respectively. E_H2_ is the energy of a hydrogen molecule adsorbed as defined previously. The energy required for successive additions of H_2_ molecules was evaluated using Eq. (2). Physically, it is used to evaluate the reversibility of storing H_2_ molecules from n to n − 1 H_2_. Correspondingly, the calculated ΔE based on GGA-PBE calculations are summarized in Table S1 with the entire ΔE larger than 0.166 eV/H_2_, our simulations confirm that the maximum adsorption numbers of H_2_ molecules can reach five for Sc as stipulated earlier. Moreover, as shown in Fig. 1(c), the featured HOMO of the first H_2_ molecule with the adsorbent indicates strong chemical adsorption between the first H_2_ molecule and Sc atom where the adsorbed H_2_ remain molecular.

### H_2_ adsorption of Ti decorated 4ND-CN_x_NT

**Figure 2:**
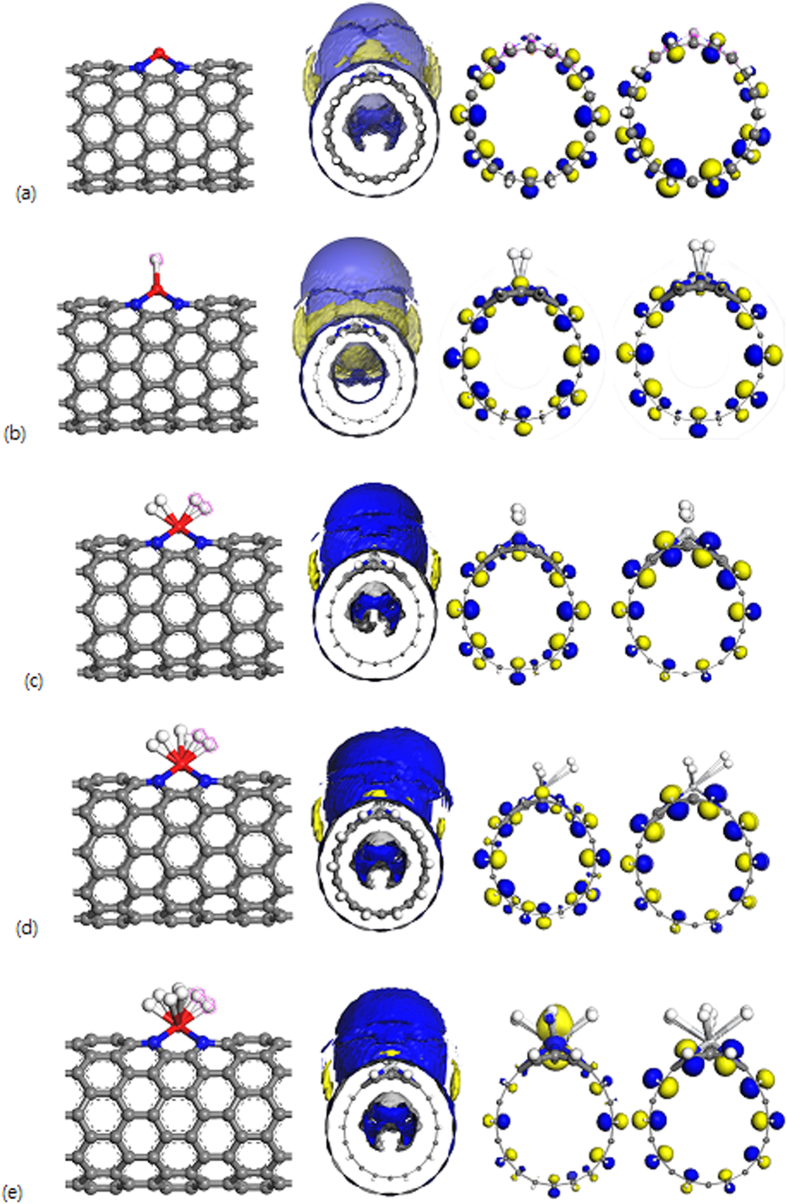
Structure, Surface Electrostatic Potential Map (SEP) and HOMO-LUMO of the optimized (**a**) Ti/4ND-CN_x_NT and Ti/4ND-CN_x_NT with (**b**) H_2_, (**c**) 2H_2_, (**d**) 3H_2_, (**e**) 4H_2_ systems. Gray color depicts Carbon; blue is Nitrogen; red is Titanium and white is Hydrogen.

Figure 2(b) shows the structural variation from spin polarized calculations as a single H_2_ molecule approaches Ti/4ND-CN_x_NT system. As in the case of Sc single adsorption, the energy always decreases first slowly and later very rapidly, at which point the hydrogen molecules are strongly attached to the Ti/4ND-CN_x_NT system. The optimized Ti-H distance is 1.920 Å. The analysis on the Ti-H_2_ distance reveals that the first added H_2_ molecule keeps a closer distance to the Ti compared to the Sc atom. The H-H bond length significantly increased from 0.752 Å of a free H_2_ to as high as 0.828 Å for Ti case. The spin polarized calculation lowers the total energy with respect to non-spin-polarized calculations and yields a triplet magnetic ground state (i.e., S = 1) for the initial Ti/4ND-CN_x_NT and the H_2_ system. However, once the hydrogen molecule is attached to Ti/4ND-CN_x_NT, the system is nonmagnetic and spin-polarized calculations are not necessary. The energy gained by the first adsorption is 0.608 eV/H_2_ much higher compared to the Sc/4ND-CN_x_NT with a value of 0.279 eV/H_2_. It is found that after the adsorption for the first H_2_ the Ti still has a 0.429 e charge, indicating that more H_2_ molecules can be absorbed. The second to forth H_2_ molecule for Ti atom gains energies within 0.211–0.332 eV per H_2_, and they are adsorbed around the Ti as shown in Fig. 2(e). The energy gained by the fourth adsorption for Ti, 0.162 (0.211 with vdW incorporated) eV/H_2_, is slightly smaller than for the other cases but is still substantial as summarized in Table S1 along with the succeeding three hydrogen molecules as they approach the Ti atom attached to its first hydrogen. For the three succeeding H_2_ attached to Ti the bond length is also elongated (0.799, 0.786, 0.767 Å). For the complete structural parameters please refer to Figure S2. The Kubas interaction is dominated by σ-donation from the H_2_ to the metal, but is more balanced between σ-donation and π-back-donation for the Ti analogues. This behavior can be traced to a lowering in energy of the metal 3d orbitals. Several attempts to add a 5^th^ hydrogen molecule at a variety of positions failed for Titanium, suggesting a limit of 4H_2_ per Ti. The final-optimized structures shown need not be the global minimum. Among many different isomers tried, a very symmetric configuration was found.

In contrast, the E_ads_ of Ti with the first H_2_ at 0.608 eV is well above the threshold while the succeeding ΔE of the second to the forth H_2_ is quite low which impedes storage reversibility of H_2_ molecules compared to Sc/4ND-CN_x_NT system. The relationship between E_ads_ and ΔE can now be established as seen clearly in Table S1 and S2 for example if we take the average of ΔE_1_ and ΔE_2_ (0.608 and 0.056 eV) it yields the E_ads_ (0.332 eV) for the two H_2_ bonded to the Ti/4ND-CN_x_NT. If we take the average of ΔE_1_, ΔE_2_ and ΔE_3_ (0.608, 0.056 and 0.105 eV) it yields the E_ads_ (0.256 eV) for the three H_2_ bonded to the Ti/4ND-CN_x_NT and so on. Therefore E_ads_ is equal to the average of ΔE_n_’s for n H_2_ bonded to the Ti/4ND-CN_x_NT. One of the major obstacles to the widespread adoption of hydrogen as a fuel is the lack of a way to store hydrogen with sufficient gravimetric and volumetric densities to be economically practical. An appealing approach for solving this problem is the development of materials that are capable of reversibly storing hydrogen at high densities. Ti/4ND-CN_x_NT store hydrogen too strongly due to the strong interaction between hydrogen and Ti metal, hindering the extraction of hydrogen under practical operating conditions. It can be noted though that the adsorption energies, E_ads,_ are still within the right range for room temperature storage for both Sc a Ti. For the number of H_2_ to be adsorbed on each metal atom, it is similar to that in Sc/Ti^-^ pure CNT or other Sc/Ti-dispersed graphitic materials, such as graphene or nanoribbons (for example, five H_2_/Sc and four H_2_/Ti)^27^,^28^,^29^,^30^,^31^,^32^. Based from previous discussions, Sc is the transition metal of choice in designing advance composite material for reversible hydrogen adsorption and desorption with promising system-weight efficiency evaluated at room temperature. While many technical issues remain to be resolved, this study will show that Sc-dispersed CNTs have great potentials for hydrogen storages.

### H_2_ Loading of (Sc/4ND)_n_-CN_x_NT

Assuming a substitutional doping through a chemical process


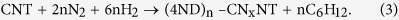


**Figure 3:**
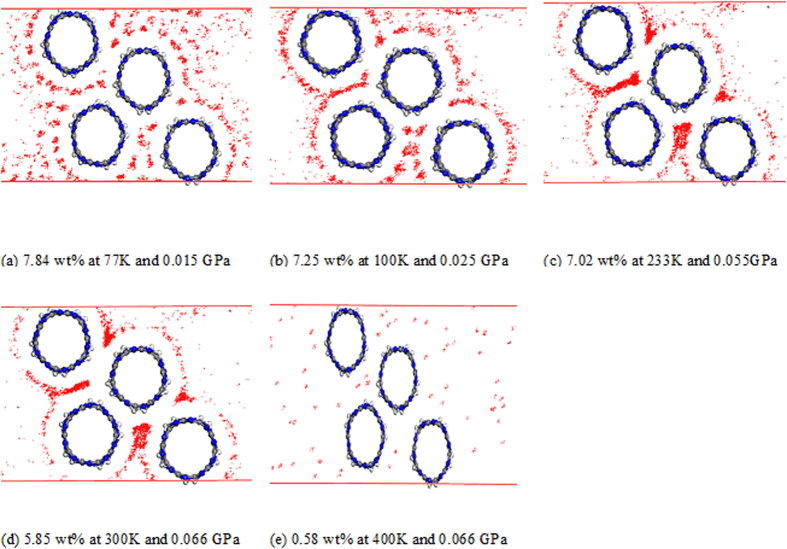
Snapshots of the adsorption configuration with the corresponding concentration in wt% between the (Sc/4ND)_10_-CN_x_NT array and H_2_ system from 77 to 400K. The adsorbed H_2_ decreases as the temperature increases.

**Figure 4:**
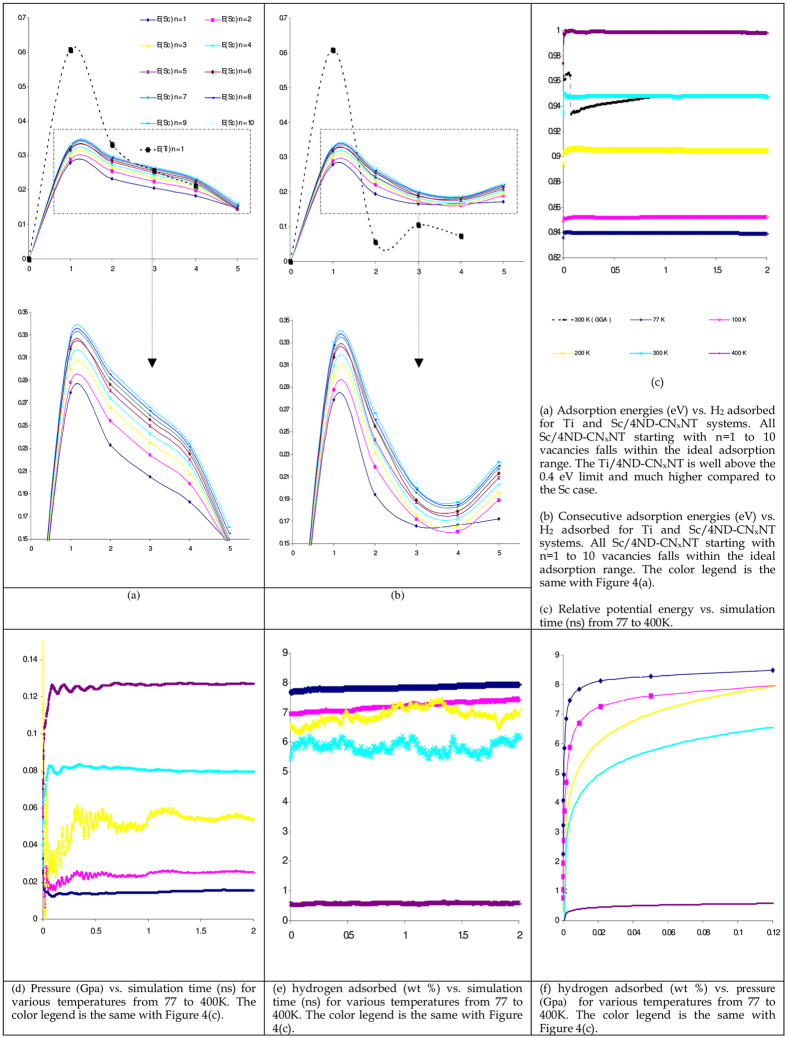
Adsorption energies, consecutive adsorption energies for Sc functionalized CN_x_NT with n = 1 to 10 4ND defects with incorporated vdW correction. H-H bond length of a free H_2_ is 0.752 Å and charge transferred from Sc to the (10, 0) CN_x_NT is 0.704 e. Relative potential energy, pressure and hydrogen adsorbed versus simulation time (ns) for various temperatures from 77 to 400 K. Magnified views of the captured areas are displayed directly below for clarity.

The formation energy of a single 4ND defect is calculated to be about 3.20 eV (endothermic) using the formula,


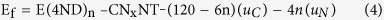


where E(4ND)_n_-CN_x_NT is the total energy of the (10, 0) 4ND-CN_x_NT with n porphyrin defects. The *u*_*C*_ is the chemical potential of C obtained from the pure CNT with 120 carbons and *u*_*N*_ is the chemical potential of N obtained from the nitrogen gas. Processes that are more realistic may be necessary for calculating the true formation energy, our estimation for n = 1 to 10 is regarded to be within the usual synthesis conditions. It is worth noting that the formation energy for a maximum of ten vacancies is 30.89 eV. A linear regression model expressed in the form of E_f_ (n) = 3.149n + 0.458 can fit the formation energy (E_f_) vs. n porphyrin defects data with r^2^ = 0.996. The physical and chemical structure of the synthesized highly nitrogen-enriched graphitic carbon was investigated experimentally by powder X-ray diffraction, scanning and transmission electron microscopy, selected area electron diffraction, energy dispersive spectroscopy, elemental analysis, Fourier transform infrared spectroscopy, X-ray photoemission spectroscopy, and electron energy loss spectroscopy and theoretically by using DFT formalisms^24^,^25^,^26^,^27^,^28^,^29^,^30^,^31^,^32^,^33^,^34^,^35^,^36^. The analysis confirmed that the product has a highly crystalline nitrogen-enriched graphitic structure with a carbon-to-nitrogen ratio of 1:1.12 corresponding to a maximum of n = 10 defects. The Sc functionalized CN_x_NT with n = 1 to 10 4ND defects was further studied. The (Sc/4ND)_10_-CN_x_NT system can be visualized by isolating a single Sc functionalized nanotube from the array shown in Fig. 3. The adsorption of H_2_ molecules with the isolated (Sc/4ND)_10_-CN_x_NT system possess favorable binding energy as seen in Table S2 as plotted in Fig. 4(a). The binding energy is considerably greater than the cohesive energy of bulk Sc metal^20^ preventing unnecessary clustering and agglomeration. As H_2_ was placed on each Sc atom, the starting configuration for each geometry optimization was taken by attaching one to five H_2_ around each n Sc atoms above the n 4ND defects, wherein the hydrogen atoms attached all remained molecular with an average bond length of around 0.755 Å. The average hydrogen adsorption energy for a (Sc/4ND)_10_-CN_x_NT complex with fifty H_2_ is 0.166 eV/H_2_ calculated using GGA-PBE functional. Therefore the H_2_ storage capacity may be predicted to be at least well above 5.8 wt%, for (Sc/4ND)_10_-CN_x_NT with 5H_2_ attached per Sc. In addition, a consecutive adsorption energy computation suggests an ideal reversible hydrogen adsorption and desorption energy range of 0.16 to 0.42 eV/H_2_ as shown in Fig. 4(b). It is astonishingly achieved at the GGA level with a ΔE_Ave_ value between 0.193 to 0.244 eV/H_2_. A Calculation using LDA-PWC was also carried out and the average hydrogen adsorption energy along with the consecutive adsorption energy is in good agreement within the requirement of hydrogen storage at room temperature.

As mentioned earlier Fig. 3 shows the (Sc/4ND)_10_-CN_x_NT array. Representative snapshots of the adsorption process in correspondence to the time history of potential energy, pressure and number of adsorption molecules are shown in Fig. 4(c–e). Here, the normal hydrogen adsorption process for the condition of liquid nitrogen temperature at 77 K and about 0.015 GPa is shown in Fig. 4(c–f) in blue. The hydrogen molecules rapidly intruded the surface of the (Sc/4ND)_10_-CN_x_NT assembly as shown in Figs 3(a) and 4(c–e). Here, the profile of the potential energy curve can be accounted for due to the sudden decrease of van der Waals interaction. The pressure change for 77 K was recorded in Fig. 4(d) versus simulation time in nanoseconds for the final configuration in Fig. 3(a). The amount of hydrogen attached to the scandium-nanotube assembly has reached 7.84 wt%. Interestingly, at 300 K the COMPASS prediction agrees well with the GGA predicted value at 5.85 wt% (Fig. 3(d)). The variations of bond lengths which is the defined to be the difference between the current length of the bond and its equilibrium value as time elapses is further analyzed. The variation of the Sc-N, C-C and H-H molecules bond length at room temperature is oscillating between 0.1 and −0.1 Å. Indicating that the entire complex is stable at room temperature. The suggested temperature for H_2_ delivery by the DOE is in the range of 233–393 K. Hence MD simulations for the maximum number of H_2_ molecules adsorbed on the surface were performed upto 400 K. It is worth noting that at 233 K the adsorb hydrogen is 7.02 wt% which is also above the DOE target. However, the (Sc/4ND)_10_-CN_x_NT arrays poorly adsorbs hydrogen at a temperature near 393 K as depicted in Fig. 3(e). This is because the hydrogen molecules have enough kinetic energy to overcome the adsorption potential of the system with temperature greater than 400 K in the range of pressure that were studied. The number of absorbed hydrogen molecules almost logarithmically increases as the pressure increases at a given temperature as seen in Fig. 4(f). The results reach the gravimetric density of DOE target, which means that the adsorption storage of hydrogen on (Sc/4ND)_10_-CN_x_NT has practical importance.

## Conclusion

The electronic properties of single-walled carbon nanotubes in the presence of 4ND defect and Sc/Ti impurities were studied using DFT. The 4ND defects in single-wall carbon nanotubes caused an enhanced chemical functionalization of Sc/Ti species suggesting a considerable reduction of clustering of metal atoms over the metal decorated nanotube. Strong binding of hydrogen molecule to the composite material Sc or Ti/4ND-CN_x_CNT was observed. The binding energy of Ti exceeded the threshold for storage reversibility of H_2_ molecules. The interaction of hydrogen molecule to the composite material was further investigated by attaching 50H_2_ around the (Sc/4ND)_10_-CN_x_CNT. It turns out that the system is capable of reversibly storing hydrogen at high densities calculated based on LDA and GGA level. Also, Molecular Dynamics simulation shows that the total excess adsorption of hydrogen on the (Sc/4ND)_10_-CN_x_NT arrays is stable with a value of 5.85 wt%. The number of absorbed hydrogen molecules almost logarithmically increases as the pressure increases at a given temperature as predicted using central force fields. Sc/4ND-CN_x_CNT complex holds promise for the design of lightweight reversible adsorption system for H_2_ storage preferentially at room temperature.

## Additional Information

**How to cite this article**: Mananghaya, M. *et al*. Scandium and Titanium Containing Single-Walled Carbon Nanotubes for Hydrogen Storage: a Thermodynamic and First Principle Calculation. *Sci. Rep.* **6**, 27370; doi: 10.1038/srep27370 (2016).

## Supplementary Material

Supplementary Information
