# Supplementary material for: Scandium and Titanium Containing Single-Walled Carbon Nanotubes for Hydrogen Storage: a Thermodynamic and First Principle Calculation
Source: Sci Rep. 2016 Jun 15;6:27370. doi: 10.1038/srep27370 (PMC4908379; doi:10.1038/srep27370)
Supplement: Supplementary Information [file srep27370-s1.doc]

**Supplementary Information**

**Scandium and Titanium Containing Single-Walled Carbon Nanotubes for Hydrogen Storage: a Thermodynamic and First Principle Calculation**

**Michael Mananghaya**

Associate Professor, De La Salle University, 2401 Taft Avenue, 0922 Manila, Philippines

Associate Professor, DLSU STC Laguna Boulevard, LTI Spine Road Barangays Biñan and Malamig, Biñan City, Laguna, Philippines

Analysis of NAnomaterials (ANA), Division IX, National Research Council of the Philippines, General Santos Ave, Taguig, 1631 Metro Manila

**Supplementary Table S1**. The adsorption energy (E_ads_), consecutive adsorption energy (ΔE) gained by successive additions of H_2_ molecules, charge transferred from Sc to the (10,0) CN_x_NT in the presence of nH_2_ (C_Sc_-H_2_) and average H-H distance (D_H2_).

| Model | E_ads_ | ΔE  (eV) | C_Sc_-H_2_  (e) | D_H2_  (Å) |
| --- | --- | --- | --- | --- |
| Sc/4ND-CN_x_NT | - | - | 0.704 (0.704*) | - |
| H_2_/Sc/4ND-CN_x_NT | 0.239 (0.279*) | 0.239 (0.279*) | 0.573 (0.567*) | 0.766 |
| (H_2_)_2_/Sc/4ND-CN_x_NT | 0.178 (0.233*) | 0.117 (0.194*) | 0.549 (0.549*) | 0.758 |
| (H_2_)_3_/Sc/4ND-CN_x_NT | 0.152 (0.205*) | 0.101 (0.166*) | 0.527 (0.523*) | 0.757 |
| (H_2_)_4_/Sc/4ND-CN_x_NT | 0.134 (0.183*) | 0.115 (0.167*) | 0.539 (0.527*) | 0.755 |
| (H_2_)_5_/Sc/4ND-CN_x_NT | 0.122 (0.146*) | 0.118 (0.172*) | 0.525 (0.520*) | 0.755 |
|  |  |  |  |  |
| Ti/4ND-CN_x_NT | - | - | 0.517 (0.525*) | - |
| H_2_/Ti/4ND-CN_x_NT | 0.536 (0.608*) | 0.536 (0.608*) | 0.418 (0.429*) | 0.828 |
| (H_2_)_2_/Ti/4ND-CN_x_NT | 0.302 (0.332*) | 0.059 (0.056*) | 0.365 (0.378*) | 0.799 |
| (H_2_)_3_/Ti/4ND-CN_x_NT | 0.209 (0.256*) | 0.023 (0.105*) | 0.350 (0.363*) | 0.786 |
| (H_2_)_4_/Ti/4ND-CN_x_NT | 0.162 (0.211*) | 0.022 (0.073*) | 0.361 (0.382*) | 0.767 |

*Incorporated with vdW, the structural parameter such as bond lengths are not affected with the correction.

mikemananghaya@gmail.com

**Supplementary Table S2**. The formation of CN_x_NT with 4ND defects (E_f_) and binding energy of nSc to (4ND)_n_-CN_x_NT denoted as E_b_. The average adsorption energy (E_ads_), consecutive adsorption energy (ΔE) gained by successive additions of H_2_ molecules, charge transferred from Sc to the (10,0) 4ND-CN_x_NT in the presence of nH_2_ (C_Sc_-_H2_) H-H distance (D_H2_) for Sc functionalized CN_x_NT with n=1 to 10 4ND defects with incorporated vdW correction. H-H bond length of a free H_2_ is 0.752 Å and charge transferred from Sc to the (10,0) CN_x_NT is 0.704 e.

| Model | E_f_  (eV) | E_b_  (eV) | E_ads_  1^st^ H_2_  (eV) | E_ads_  2^nd^ H_2_  (eV) | E_ads_  3^rd^ H_2_  (eV) | E_ads_  4^th^ H_2_  (eV) | E_ads_  5^th^ H_2_  (eV) | E_Ave_  (eV) | C_(Sc_-_H2)Ave_  (e) | D_(H2) Ave_  (Å) |
| --- | --- | --- | --- | --- | --- | --- | --- | --- | --- | --- |
|  |  |  |  |  |  |  |  |  |  |  |
| Sc/4ND-CN_x_NT | 3.200 | 8.770 | 0.279 | 0.233 | 0.205 | 0.183 | 0.146 | 0.209 | 0.537 | 0.758 |
| (Sc/4ND)_2_-CN_x_NT | 4.651 | 8.602 | 0.288 | 0.254 | 0.224 | 0.199 | 0.143 | 0.222 | 0.540 | 0.758 |
| (Sc/4ND)_3_-CN_x_NT | 9.452 | 8.244 | 0.300 | 0.266 | 0.235 | 0.208 | 0.143 | 0.231 | 0.539 | 0.758 |
| (Sc/4ND)_4_-CN_x_NT | 12.590 | 8.121 | 0.309 | 0.274 | 0.243 | 0.215 | 0.143 | 0.237 | 0.533 | 0.757 |
| (Sc/4ND)_5_-CN_x_NT | 14.350 | 8.069 | 0.319 | 0.281 | 0.250 | 0.220 | 0.145 | 0.243 | 0.521 | 0.757 |
| (Sc/4ND)_6_-CN_x_NT | 17.590 | 8.059 | 0.317 | 0.286 | 0.255 | 0.225 | 0.147 | 0.246 | 0.513 | 0.756 |
| (Sc/4ND)_7_-CN_x_NT | 22.010 | 7.906 | 0.325 | 0.291 | 0.259 | 0.228 | 0.150 | 0.251 | 0.493 | 0.756 |
| (Sc/4ND)_8_-CN_x_NT | 25.150 | 7.882 | 0.328 | 0.295 | 0.263 | 0.231 | 0.155 | 0.254 | 0.472 | 0.756 |
| (Sc/4ND)_9_-CN_x_NT | 28.290 | 7.891 | 0.331 | 0.299 | 0.266 | 0.234 | 0.160 | 0.258 | 0.448 | 0.755 |
| (Sc/4ND)_10_-CN_x_NT | 30.890 | 7.890 | 0.332 | 0.300 | 0.268 | 0.237 | 0.166 | 0.261 | 0.419 | 0.755 |
|  |  |  |  |  |  |  |  |  |  |  |

| Model | ΔE  1^st^ H_2_  (eV) | ΔE  2^nd^ H_2_  (eV) | ΔE  3^rd^ H_2_  (eV) | ΔE  4^th^ H_2_  (eV) | ΔE  5^th^ H_2_  (eV) | ΔE_Ave_  (eV) |
| --- | --- | --- | --- | --- | --- | --- |
|  |  |  |  |  |  |  |
| Sc/4ND-CN_x_NT | 0.279 | 0.194 | 0.166 | 0.167 | 0.172 | 0.193 |
| (Sc/4ND)_2_-CN_x_NT | 0.288 | 0.219 | 0.172 | 0.161 | 0.189 | 0.206 |
| (Sc/4ND)_3_-CN_x_NT | 0.300 | 0.232 | 0.175 | 0.166 | 0.196 | 0.214 |
| (Sc/4ND)_4_-CN_x_NT | 0.309 | 0.240 | 0.182 | 0.172 | 0.203 | 0.221 |
| (Sc/4ND)_5_-CN_x_NT | 0.319 | 0.243 | 0.187 | 0.176 | 0.209 | 0.227 |
| (Sc/4ND)_6_-CN_x_NT | 0.317 | 0.255 | 0.189 | 0.179 | 0.213 | 0.231 |
| (Sc/4ND)_7_-CN_x_NT | 0.325 | 0.256 | 0.196 | 0.183 | 0.217 | 0.235 |
| (Sc/4ND)_8_-CN_x_NT | 0.328 | 0.261 | 0.199 | 0.185 | 0.220 | 0.239 |
| (Sc/4ND)_9_-CN_x_NT | 0.331 | 0.267 | 0.200 | 0.187 | 0.223 | 0.242 |
| (Sc/4ND)_10_-CN_x_NT | 0.332 | 0.269 | 0.205 | 0.190 | 0.225 | 0.244 |
|  |  |  |  |  |  |  |

| (a)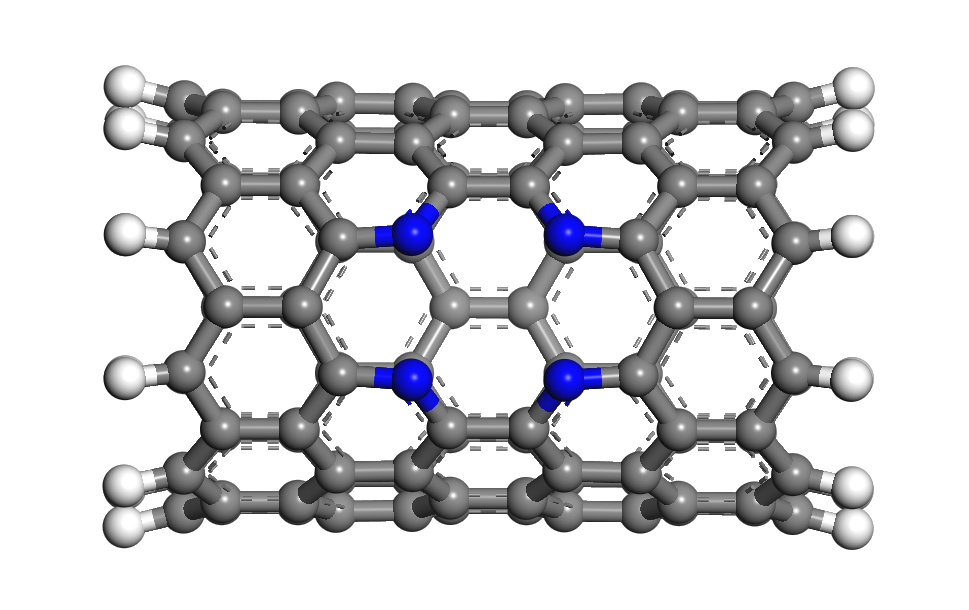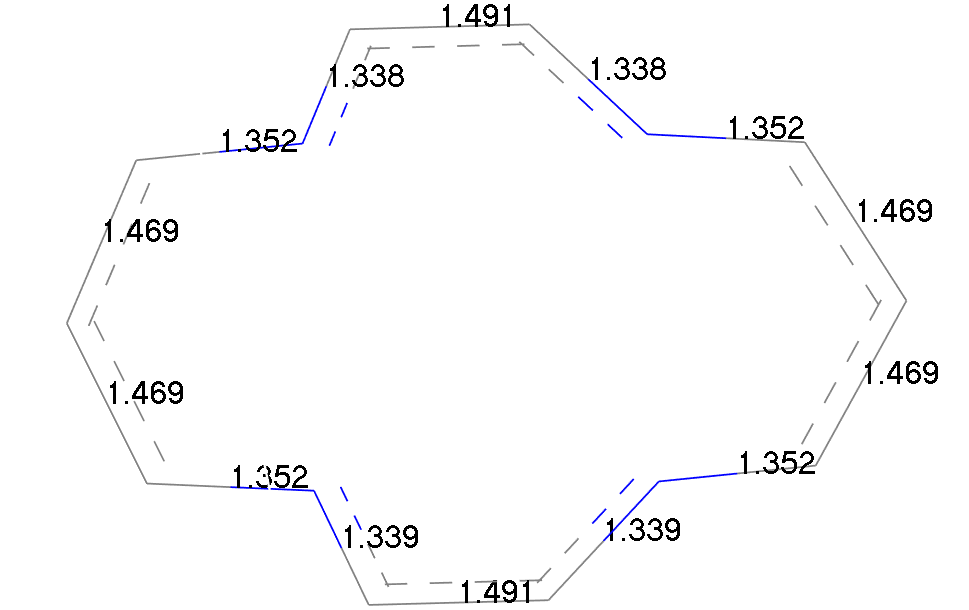(b)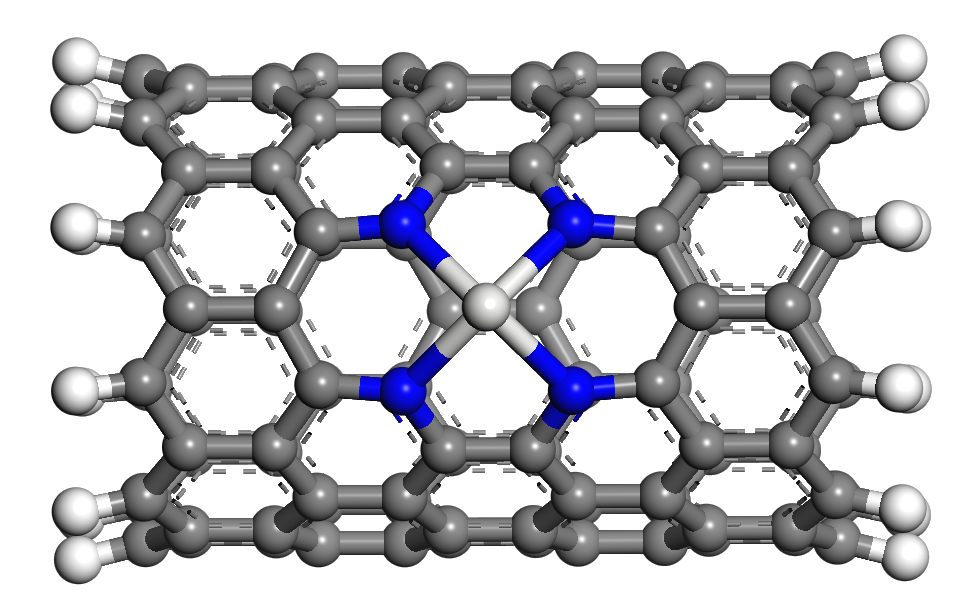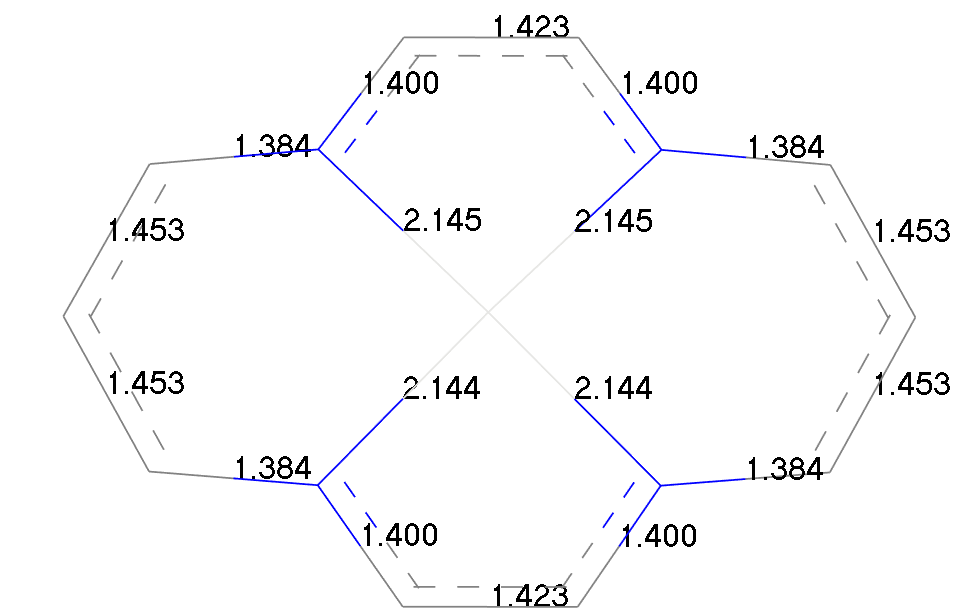 |
| --- |
| (c)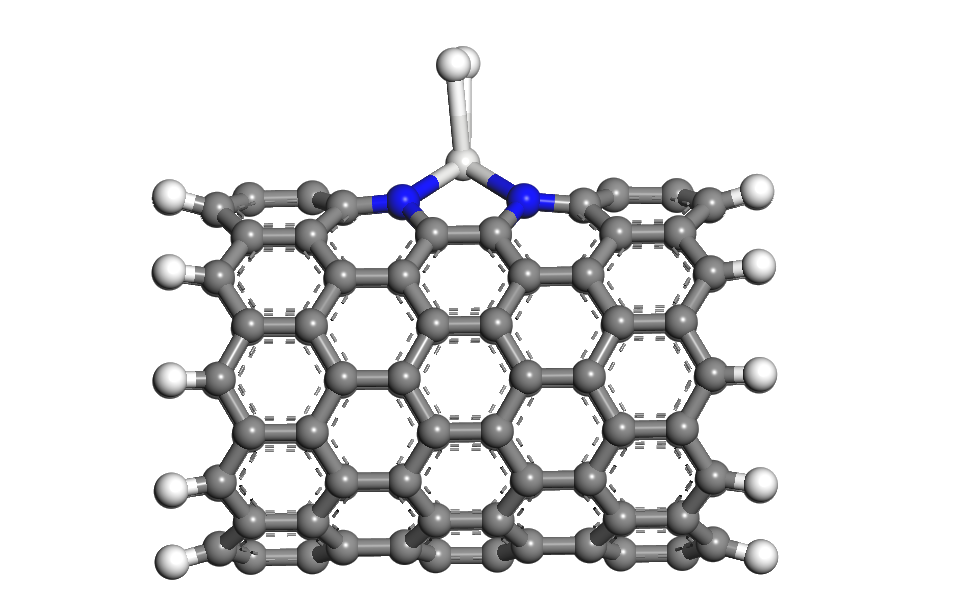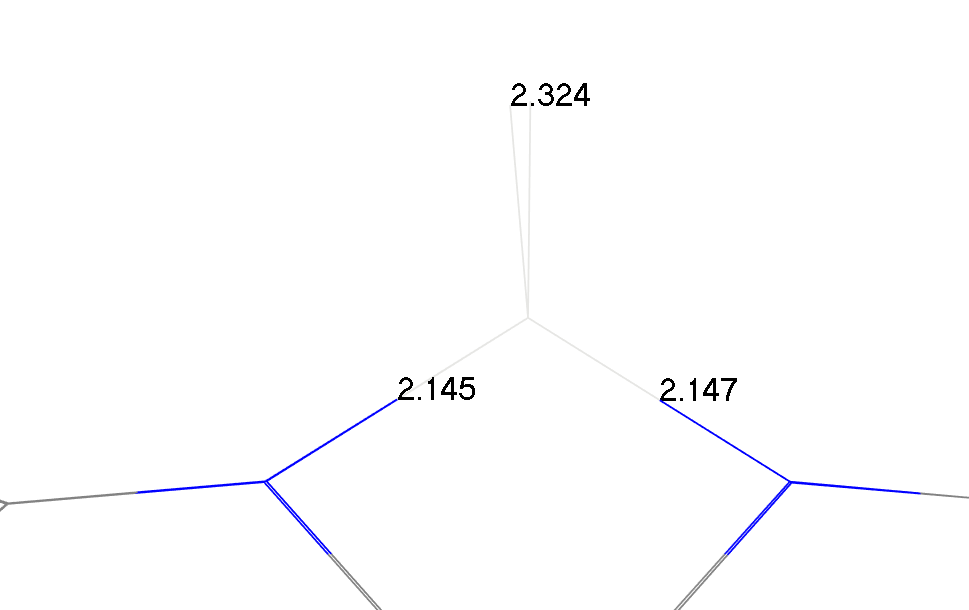(d) 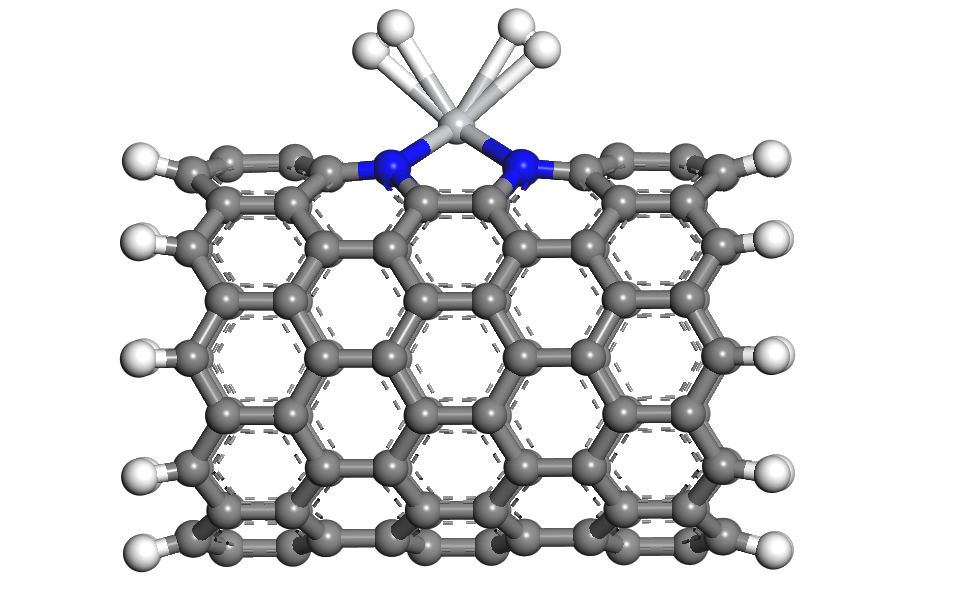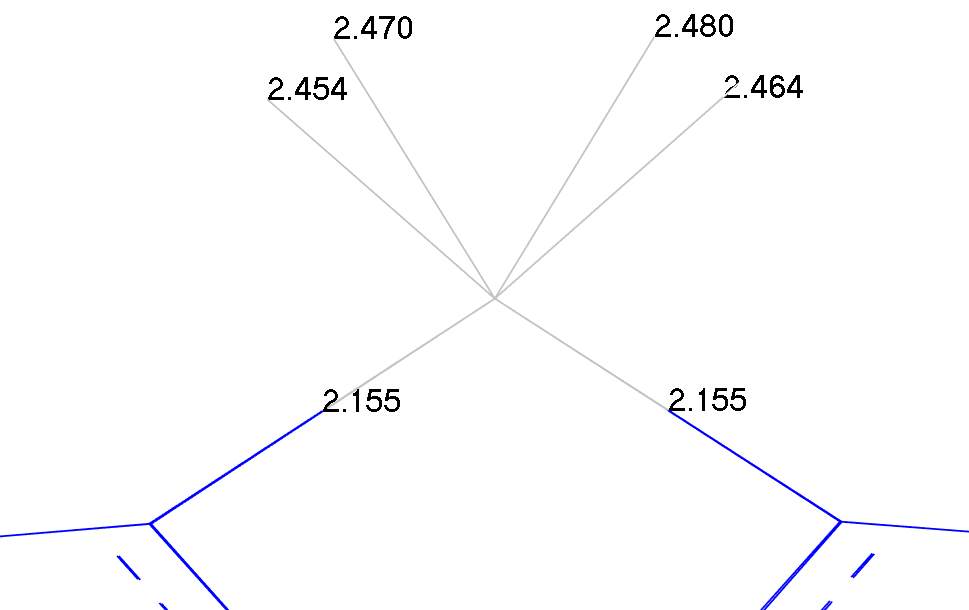 |
| (e)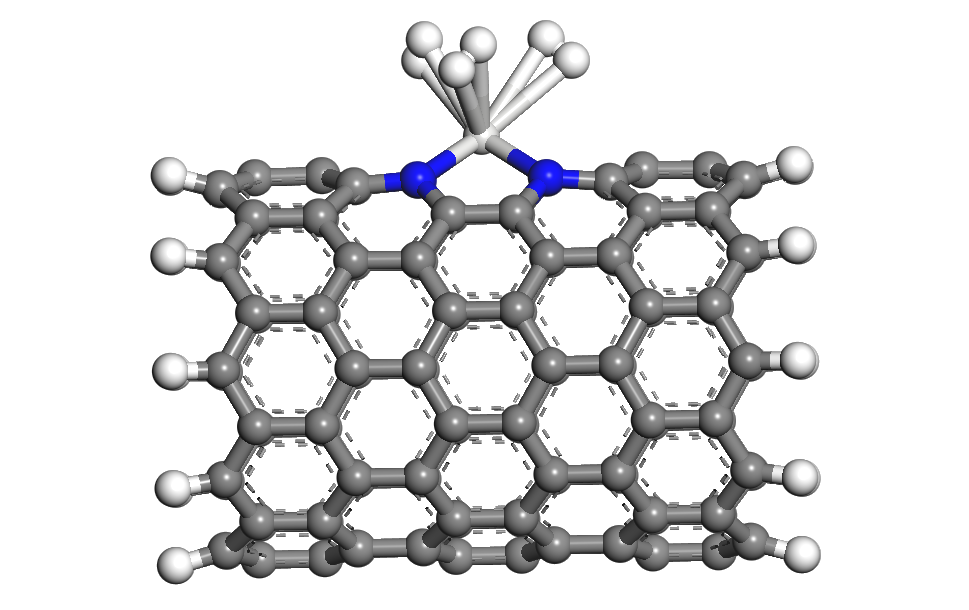 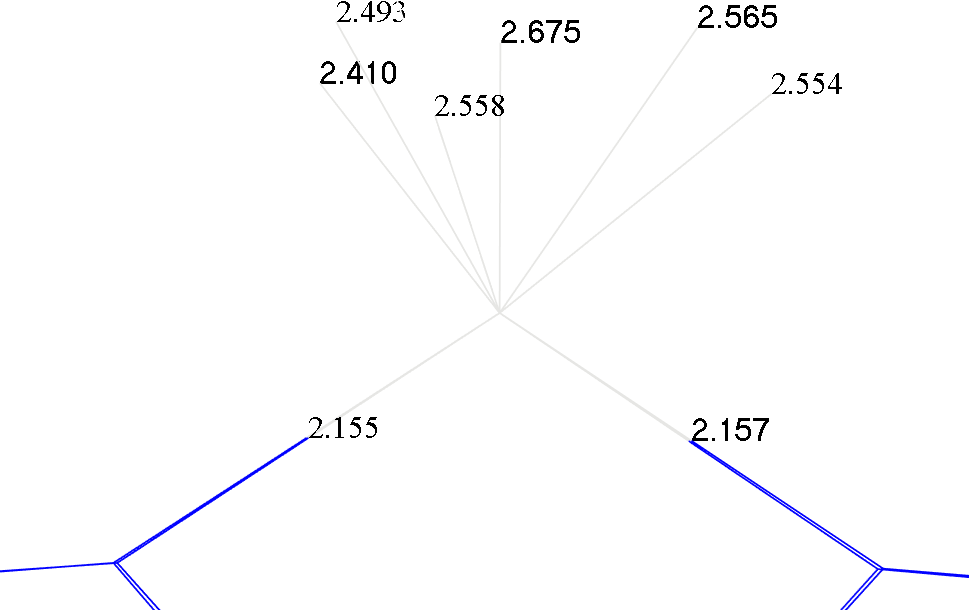 (f)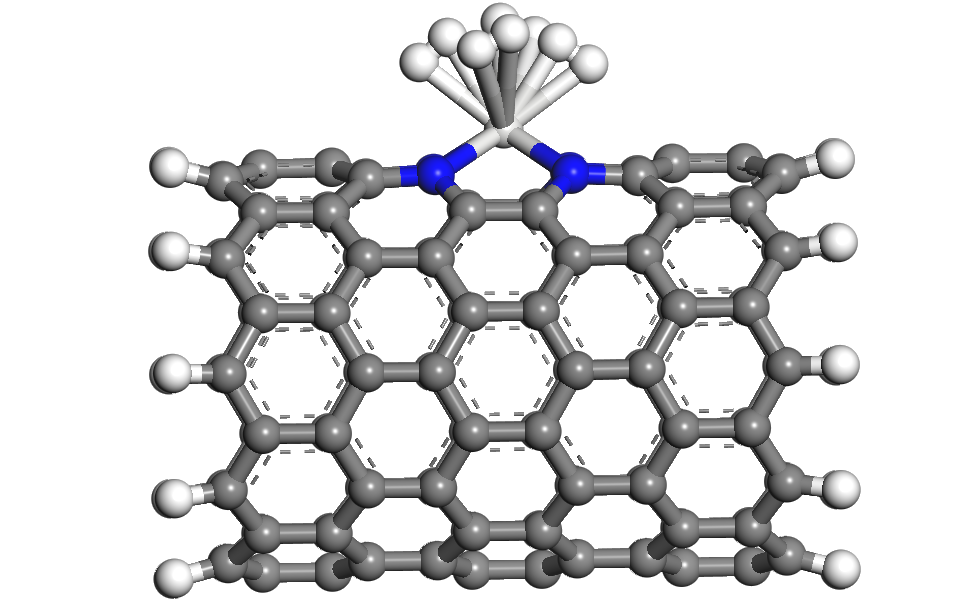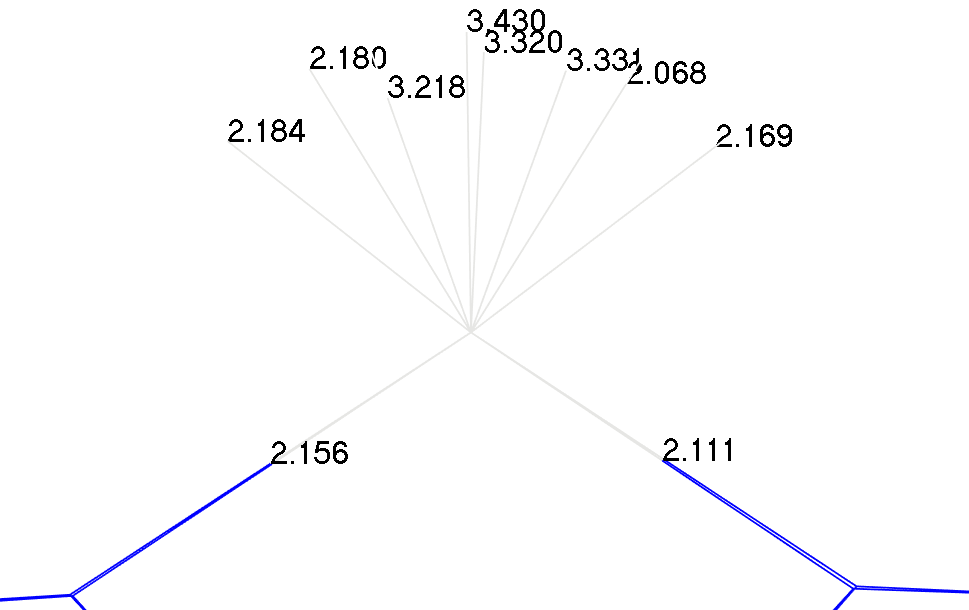 |
| (g)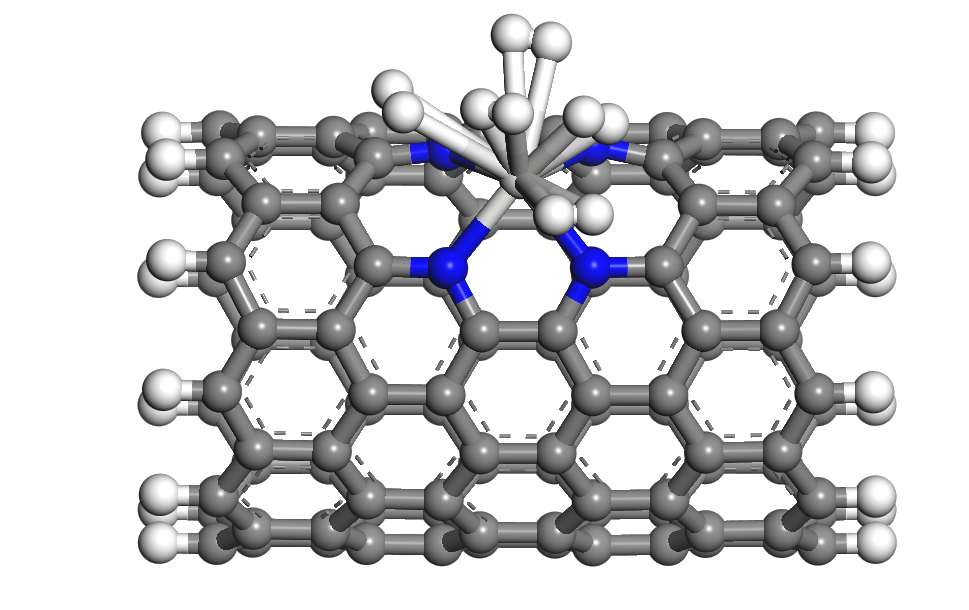 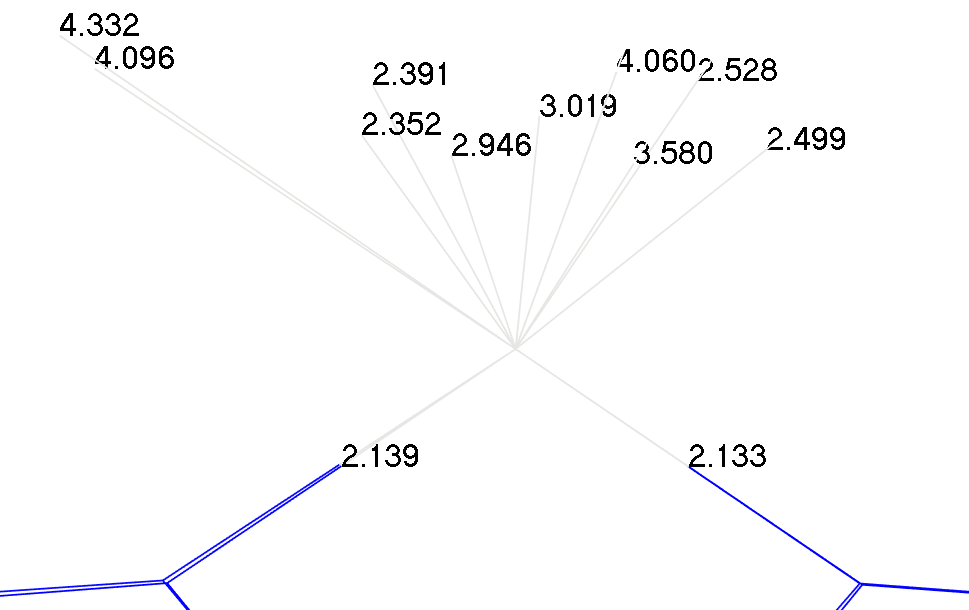 |

**Supplementary Figure S1.** Magnified view of the structural parameters and bond lengths of the optimized (a) 4ND-CN_x_NT, (b) Sc/4ND-CN_x_NT and Sc/4ND-CN_x_NT with (c) H_2_, (d) 2H_2_, (e) 3H_2_ , (f) 4H_2_, (g) 5H_2_ systems.

| (a) 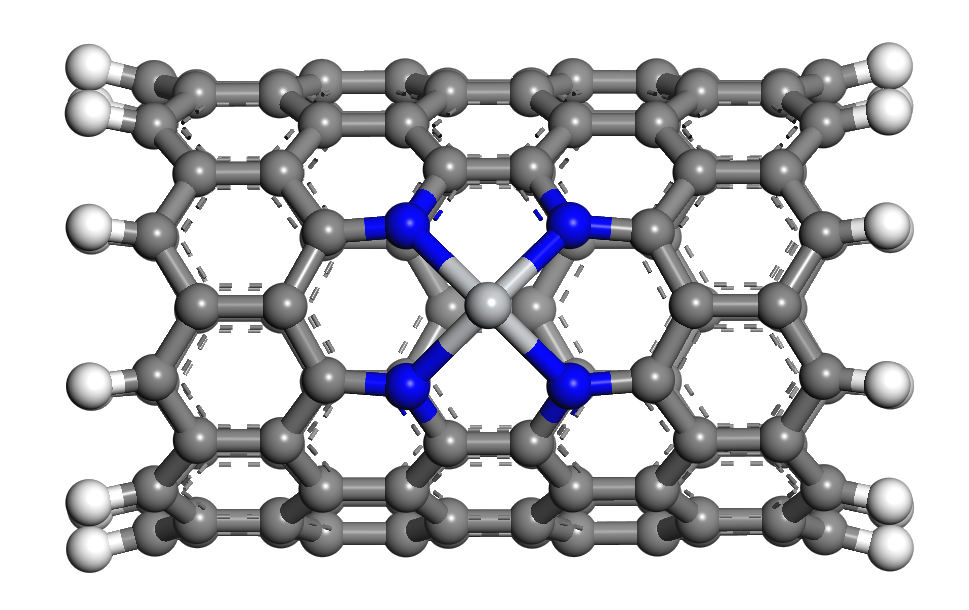 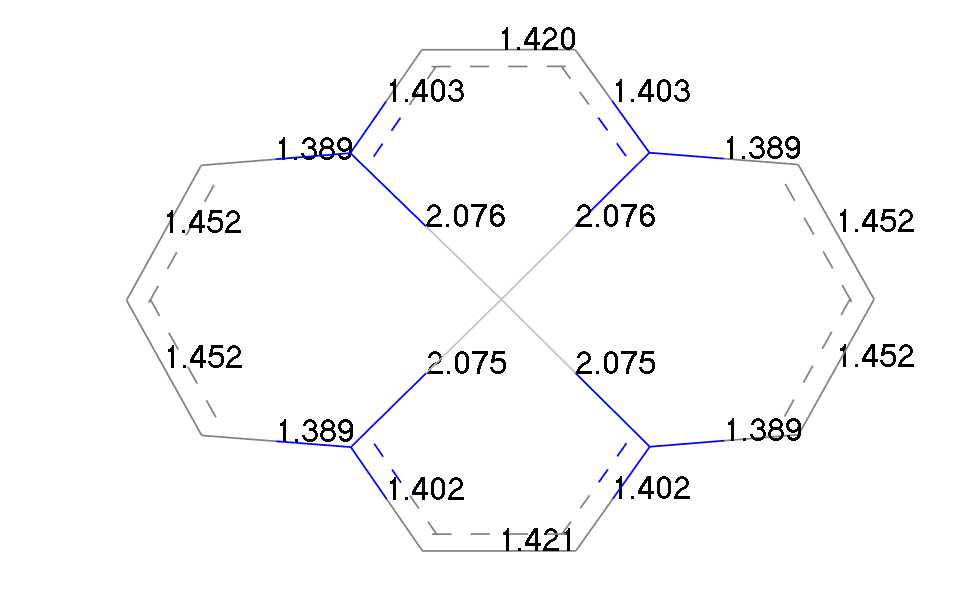 |
| --- |
| (b)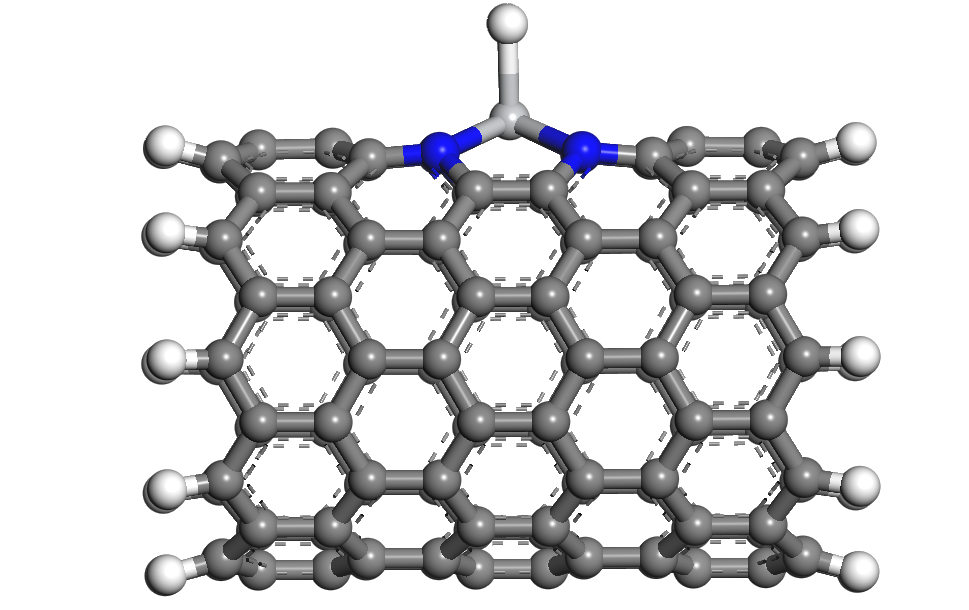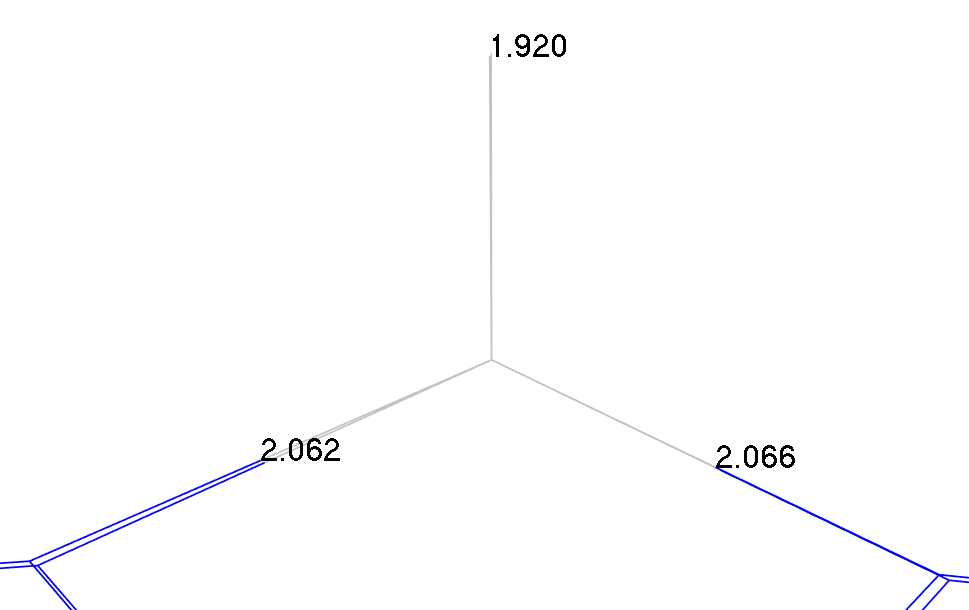 (c)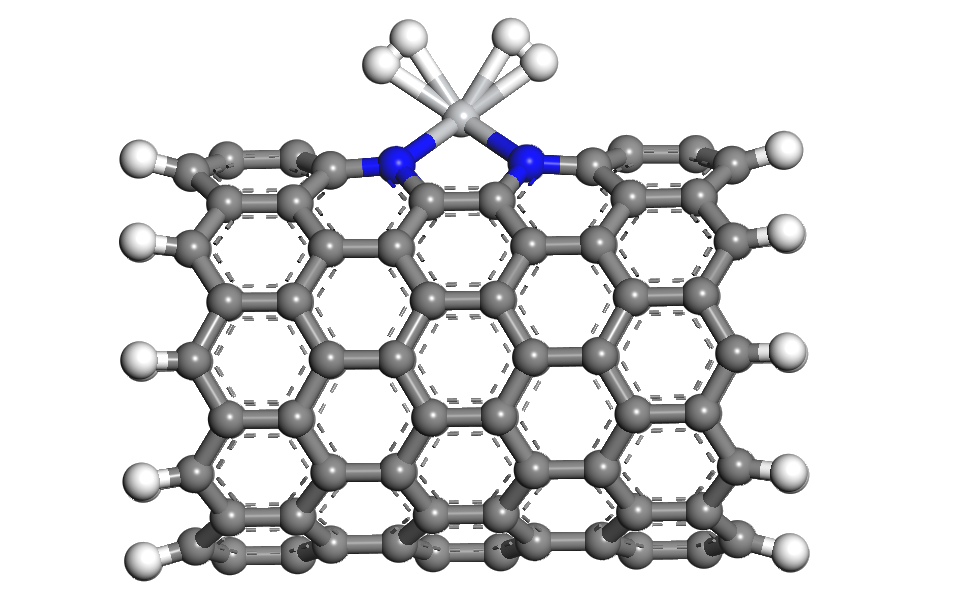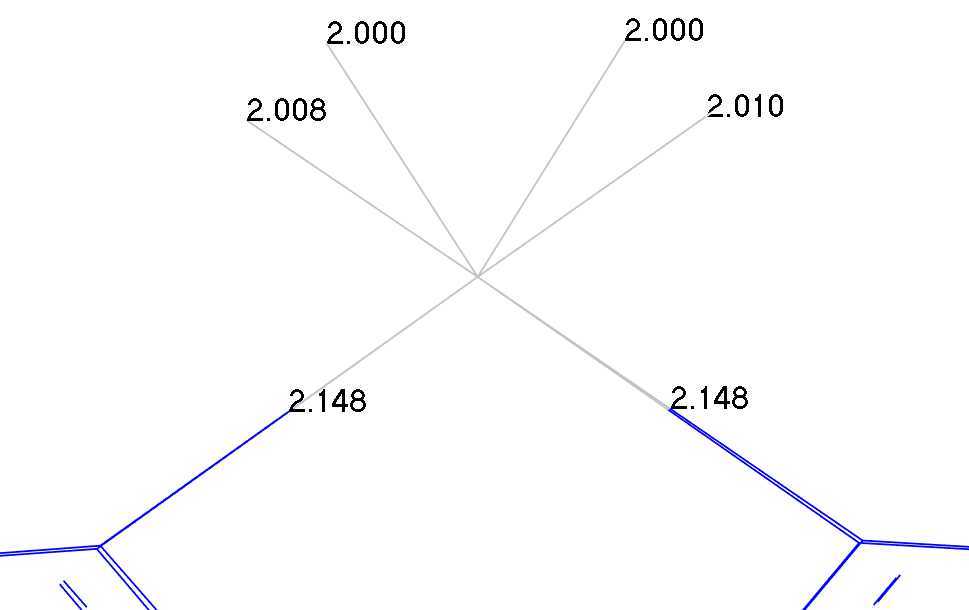 |
| (d)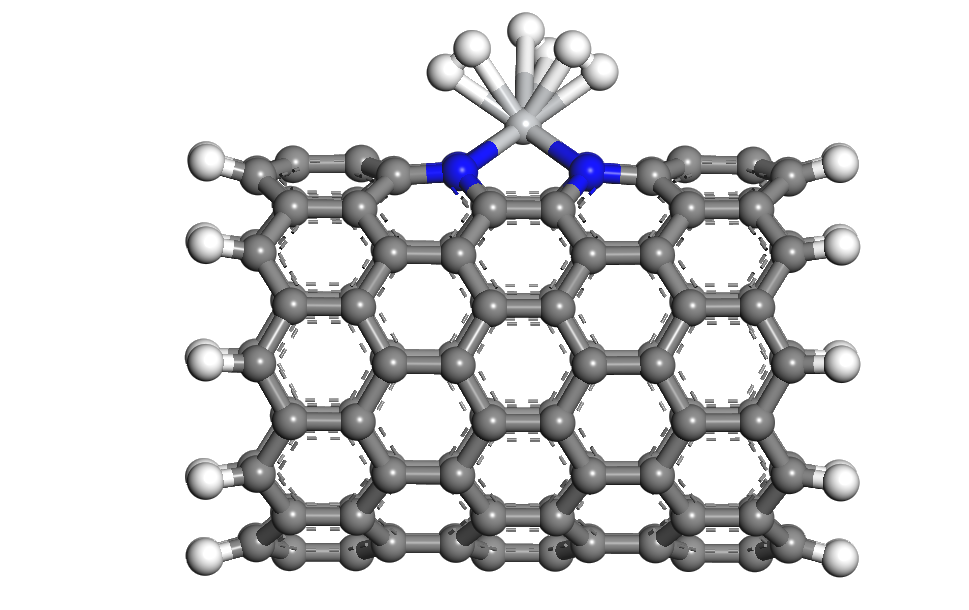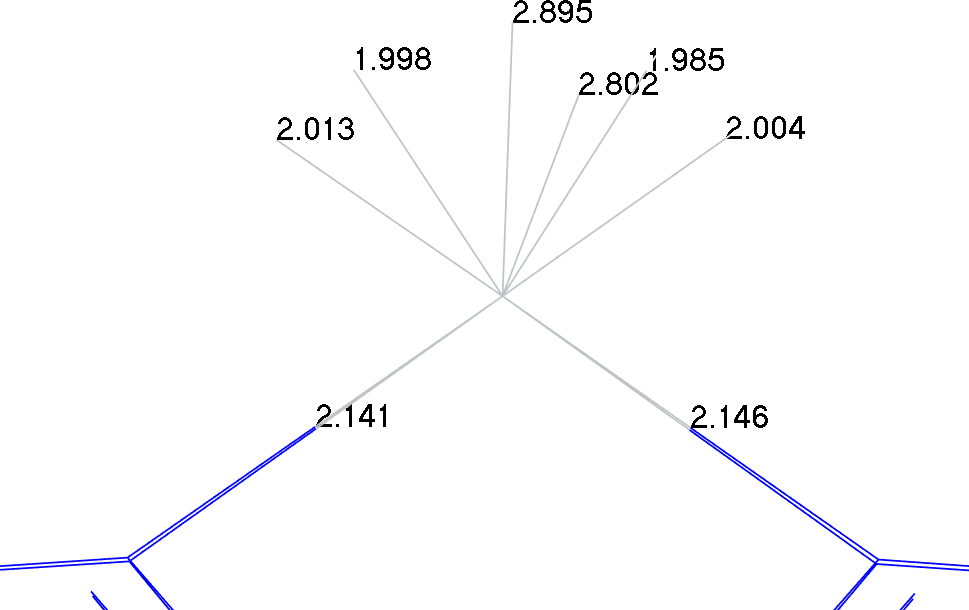(e)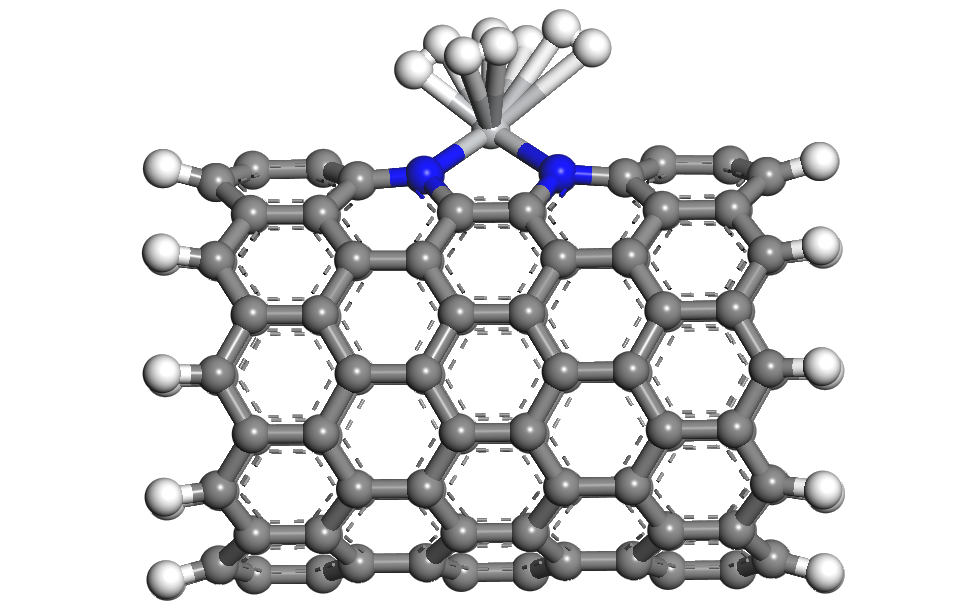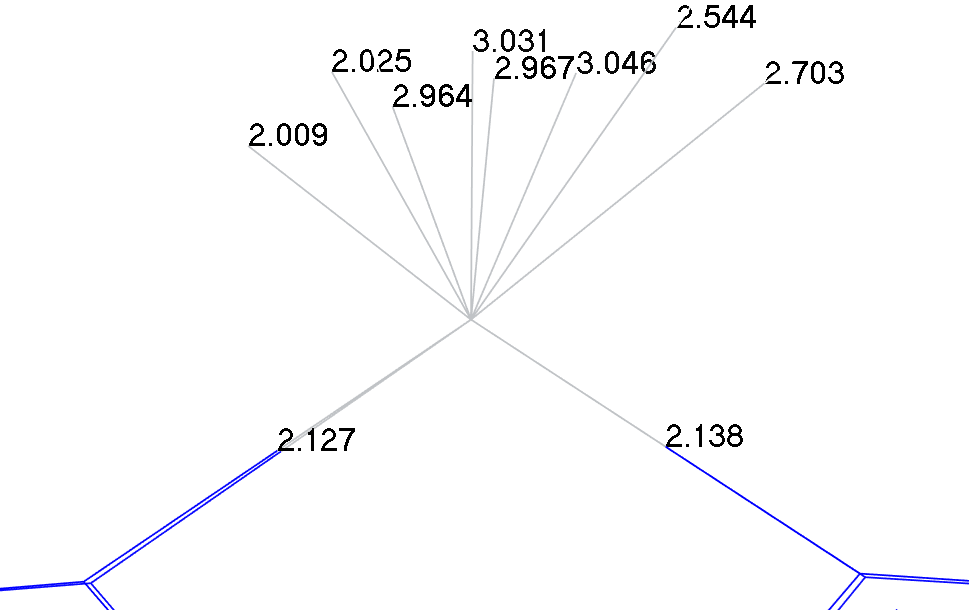 |

**Supplementary Figure S2.** Magnified view of the structural parameters and bond lengths of the optimized (a) Ti/4ND-CN_x_NT and Ti/4ND-CN_x_NT with (b) H_2_, (c) 2H_2_, (d) 3H_2_ , (e) 4H_2_ systems.
